# A New Mechanically‐Interlocked [Pd_2_L_4_] Cage Motif by Dimerization of two Peptide‐based Lemniscates

**DOI:** 10.1002/anie.202010995

**Published:** 2020-10-13

**Authors:** Thorben R. Schulte, Julian J. Holstein, Laura Schneider, Abdulselam Adam, Gebhard Haberhauer, Guido H. Clever

**Affiliations:** ^1^ Faculty of Chemistry and Chemical Biology TU Dortmund Univ. Otto-Hahn-Str. 6 44227 Dortmund Germany; ^2^ Institute for Organic Chemistry Univ. Duisburg-Essen Universitätsstr. 7 45117 Essen Germany

**Keywords:** catenane, chirality, coordination cage, mechanical bond, supramolecular chemistry

## Abstract

Most metallo‐supramolecular assemblies of low nuclearity adopt simple topologies, with bridging ligands spanning neighboring metal centers in a direct fashion. Here we contribute a new structural motif to the family of host compounds with low metal count (two) that consists of a pair of doubly‐interlocked, Figure‐eight‐shaped subunits, also termed “lemniscates”. Each metal is chelated by two chiral bidentate ligands, composed of a peptidic macrocycle that resembles a natural product with two pyridyl‐terminated arms. DFT calculation results suggest that dimerization of the mononuclear halves is driven by a combination of 1) Coulomb interaction with a central anion, 2) π‐stacking between intertwined ligand arms and 3) dispersive interactions between the structure's compact inner core bedded into an outer shell composed of the cavitand‐type macrocycles. The resulting cage‐like architecture was characterized by NMR, MS and X‐ray structure analyses. This new mechanically bonded system highlights the scope of structural variety accessible in metal‐mediated self‐assemblies composed of only a few constituents.

## Graphical abstract

A chiral macrocycle, equipped with two pyridine donors, assembles with Pd^II^ cations to an unprecedented metallo‐supramolecular architecture by pairwise catenation of two figure‐eight‐shaped subunits. Interlocking in solid state and solution is driven by close non‐covalent interaction between the basket‐shaped macrocycles and coordinating ligand arms.

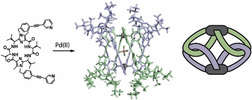

The size and structure of metallo‐supramolecular architectures, self‐assembling from metal cations and bridging ligands under thermodynamic control, are determined by a combination of entropic and enthalpic factors.^[1]^ While the former ones are dominated by the drive to form structures of low nuclearity and the release of ordered solvents from inner voids, latter effects combine dative bonding and a variety of attractive non‐covalent contributions (i.e. Coulomb forces, hydrogen bonds, π‐stacking, dipole interactions, London dispersion) with unfavorable ring strain and repulsive effects of steric crowding. Most structures formed by these principles show rather simple topologies in which organic ligands connect neighboring metal nodes following direct paths.^[2]^ In the large family of [Pd_*n*_L_2*n*_] assemblies, consisting of square‐planar coordinated Pd^II^ cations surrounded by bis‐monodentate bridging ligands, this trend and its exceptions can be nicely observed.^[3]^ For example, structures with *n*=3 usually form unentangled, three‐membered rings with metals sitting on the corner of an equilateral triangle, connected by three ligands from above the triangular plane and three from below. In 2012, we reported a unique variation of this principle, featuring both sets of three ligands braided into a trefoil knot.^[4]^ Structural variety in the *n*=4 class is larger, with four‐membered crowns forming the simplest morphology,^[3c]^ accompanied by tetrahedra,^[5]^ first reported by Lützen et al,^[6]^ interpenetrated double cages^[7]^ and more exotic variations such as a bridged‐pair‐of‐bowls topology.^[8]^ Examples of even larger assemblies include the recent report of a [Pd_6_L_12_] cage‐in‐ring pseudorotaxane,^[9]^ a huge [Pd_8_L_16_] Hopf Link^[5]^ and Fujita's [Pd_48_L_96_] Goldberg polyhedra^[10]^ and tethered sphere‐in‐sphere structure.^[11]^

As for members of the group with lowest nuclearity [Pd_2_L_4_], a large variety of the topologically most simple lantern‐shaped cages (Figure 1 a) were reported by Steel,^[3b]^ Fujita,^[12]^ Yoshizawa,^[13]^ Chand,[^3e^, ^14^] Crowley,[^3f^, ^15^] our group[^8^, ^16^] and others.^[17]^ Recently, we introduced the first example of a self‐penetrating [Pd_2_L_4_] topology, albeit composed of two different ligands in a heteroleptic fashion (Figure 1 b).^[18]^ Here, we present the first example of a [Pd_2_L_4_] structural motif containing both metals chelated by two bidentate donors, in which these two Figure‐eight‐shaped (“lemniscate”) subunits doubly catenate to yield a pseudo‐cage structure with a central cavity (Figure 1 c).^[19]^

**Figure 1:**
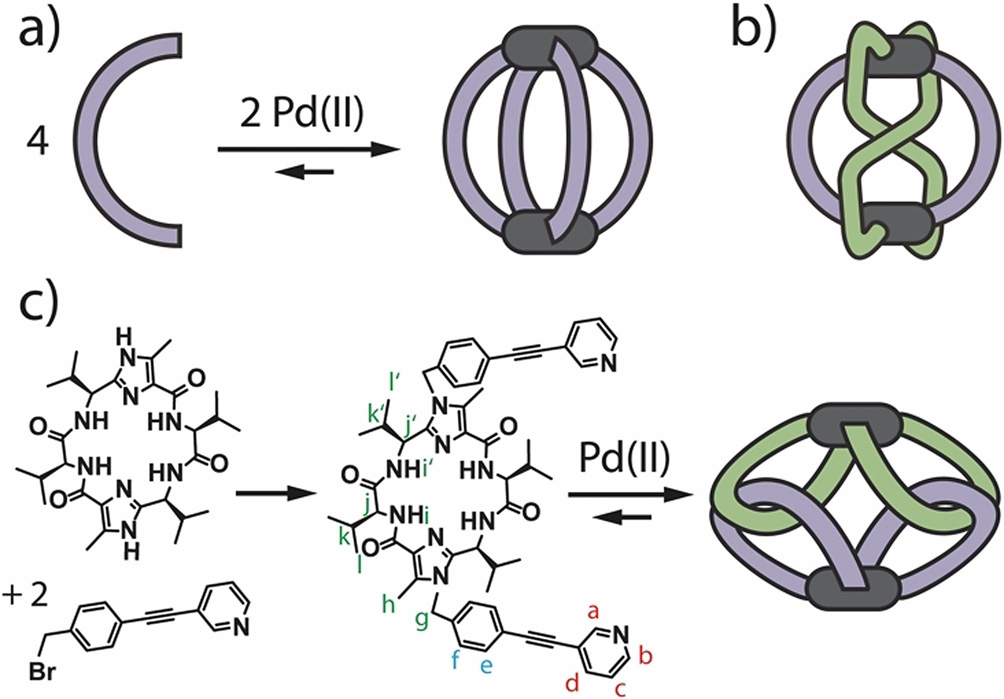
a) Pd^II^‐mediated assembly of the usual lantern‐shaped [Pd_2_L_4_] cage geometry; b) alternative self‐penetrating topology^[18]^ and c) synthesis of ligand **L** and assembly with Pd^II^ cations to an interlocked dimer of Figure‐of‐eights.

The herein used ligand was derived from a chiral peptidic macrocycle whose imidazole components were previously shown to allow modification by *N*‐alkylation.^[20]^ Hence, attachment of two pyridyl‐terminated arms yielded ligand **L** which was examined for its ability to form natural product‐inspired supramolecular structures by metal‐mediated self‐assembly. The strategy to include biomimetic elements into discrete artificial nano‐structures has recently raised some attention. In the context of Pd^II^‐based systems, Fujita and co‐workers have realized intricate metallo‐supramolecular architectures with peptide backbones.^[21]^ Natarajan et al. have assembled [Pd_2_L_4_] “cages on steroids” from cholic acid derivatives.^[22]^ We equipped both faces of DNA G‐quadruplexes with pyridine ligands, allowing to bind square‐planar Cu^II^ cations whose distance across the bio‐hybrid helicate could be determined by EPR methods.^[23]^ The integration of biocompatible motifs into artificial molecules and assemblies bears application potential for the (multitopic) binding to proteins or other biopolymers.^[24]^ Furthermore, their homochiral nature makes them prospective candidates for specific guest recognition and asymmetric catalysis.^[25]^ The chiral backbone used in this work is a macrocyclic pseudo‐peptide inspired by marine metabolites and has already been applied in the design of molecular motors^[26]^ and chiral switches.^[27]^

Ligand **L** was synthesized from previously reported macrocycle^[20]^ via nucleophilic substitution at a benzylic bromide carrying a pyridine ligand functionality. Addition of 2 equiv. of enantiopure **L** to [Pd(CH_3_CN)_4_](BF_4_)_2_ in [D_6_]DMSO afforded a compound with a simple NMR spectrum (Figure 2 a, middle) and an ESI mass spectrum (Figure 3 a) supporting formation of mononuclear complex [Pd**L**_2_]^2+^, most probably featuring a “Figure‐eight” appearance as a result of two ligands **L** chelating the square‐planar coordinated Pd^II^ cation. The same reaction carried out in CD_3_CN, monitored by ^1^H NMR spectroscopy, initially revealed convoluted signal splitting and shifting (SI Figure S7), as compared to the spectrum of the free ligand or the mononuclear [Pd**L**_2_]^2+^ compound. Pleasingly, the spectrum could be significantly simplified by addition of 1 equiv. Cl^−^ (as NBu_4_Cl) and heating at 70 °C overnight (Figure 2 a). As a result, only two signal sets, one featuring intensive signals and one of lower magnitude, remained, both showing a two‐fold splitting for every signal. Interestingly, the use of NTf_2_^−^ as counter ion in the presence of Cl^−^ resulted in an even more appealing ^1^H spectrum, showing a larger fraction of the major component. High resolution electrospray ionization (ESI) mass spectra were recorded for the tetrafluoroborate and chloride containing samples in acetonitrile, yielding strong signals for species [BF_4_@Pd_2_ **L**_4_]^3+^ and [Cl@Pd_2_ **L**_4_]^3+^, respectively (Figure 3 b,c). No signals indicating the presence of species with stoichiometries other than [Pd_2_ **L**_4_] could be found. We further applied trapped ion mobility spectrometry (TIMS) to the major ions detected in the mass spectra, revealing gas‐phase collisional cross sections (CCS) pointing to a significant size increase from the monomeric [Pd**L**_2_]^2+^ complex to the species associated with stoichiometry [BF_4_@Pd_2_ **L**_4_]^3+^ (insets in Figure 3). In line with the convoluted NMR spectra of this sample, ion mobility showed a broad distribution of at least two components with significantly deviating CCS values, probably resulting from coexisting stereoisomers and/or conformers in equilibrium. Again, chloride addition to obtain [Cl@Pd_2_ **L**_4_]^3+^ significantly simplified the spectrum, leaving one single, sharp mobility signal. Interestingly, its CCS value was found to be smaller than any of the peaks associated with stoichiometry [BF_4_@Pd_2_ **L**_4_]^3+^, indicating a contraction of the chloride‐bound form as compared to the ones containing the larger BF_4_^−^ anion. The combination of these mass spectrometric findings, together with the observed signal splitting in the NMR spectra, gave us a first indication that the assembly products formed from the enantiopure ligand may feature a more complex structure than the typical lantern‐shape object shown in Figure 1 a.

**Figure 2:**
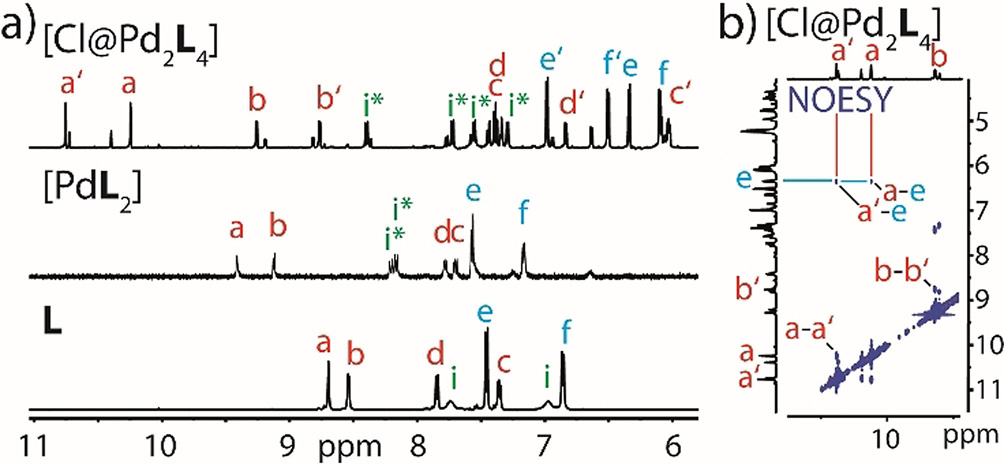
a) Partial ^1^H NMR spectra of ligand **L** (in CD_3_CN), mononuclear assembly [Pd**L**_2_] (in [D_6_]DMSO) and interlocked dimer [Cl@Pd_2_ **L**_4_] (in CD_3_CN) encapsulating a Cl^−^ anion (peaks of major isomer assigned; small peaks=minor isomer); d) partial NOESY NMR spectrum of [Cl@Pd_2_ **L**_4_] revealing characteristic contacts H_a_/_a′_ to H_e_ only observable in the catenated structure but not in the free ligand.

**Figure 3:**
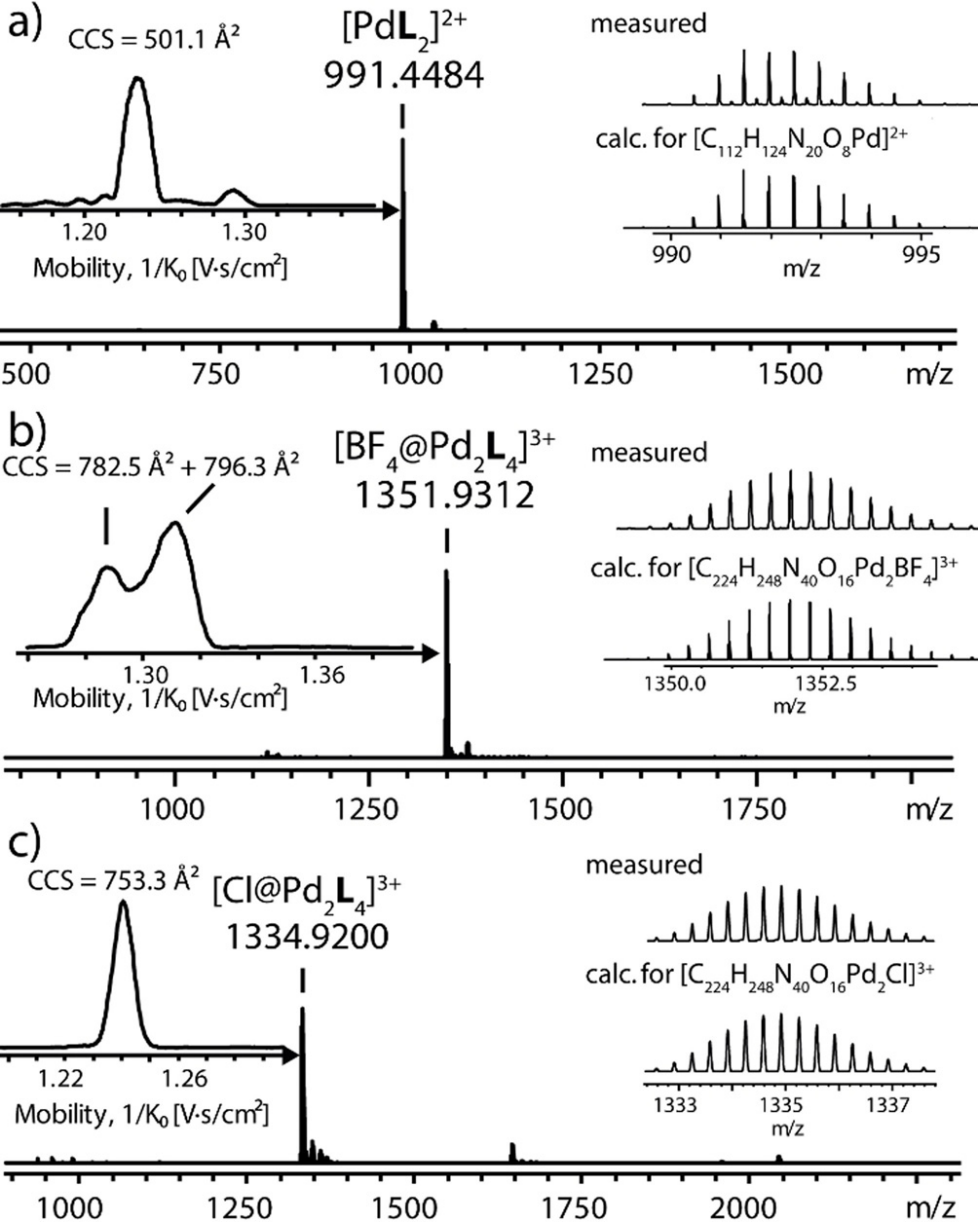
High‐resolution ESI mass and ion mobility spectra of a) mononuclear [Pd**L**_2_] (calc. 991.4483), b) tetrafluoroborate‐containing [Pd_2_ **L**_4_] (calc. 1351.9333) and c) chloride‐encapsulating [Cl@Pd_2_ **L**_4_] (calc. 1334.9216). The gas phase ion mobility values and computed collisional cross sections (CCS) reveal a size increase from the monomer to the interlocked structure, a less defined structure for the BF_4_^−^‐containing dimer (two broad mobility signals for *m*/*z=*1351.6; also compare convoluted NMR in Figure S7) and a well‐defined, again slightly contracted structure of [Cl@Pd_2_ **L**_4_].

This assumption was finally verified after obtaining a single crystal X‐ray structure. Crystals suitable for diffraction experiment were grown by slow diffusion of Et_2_O into a mixture of **L** and [Pd(CH_3_CN)_4_](BF_4_)_2_ (without addition of chloride) in acetonitrile. The compound crystallized in space group *P2_1_* with two full [Pd_2_ **L**_4_] assemblies of same topology in the asymmetric unit. The structure can be described as a catenane made of two Figure‐eight‐shaped [Pd**L**_2_] units doubly interlocked to a *C_2_*‐symmetric [Pd_2_ **L**_4_] pseudo‐cage (Figure 4 a–c). One BF_4_^−^ ion was found encapsulated in the cavity between the two Pd^II^ cations. The Pd‐Pd distance is 8.2 Å. Each Pd^II^ cation is chelated by two ligands, coordinated in *cis*‐configuration. Hence, in contrast to most known Pd^II^‐mediated architectures, the ligands do not directly bridge the metal cations. Instead it is the mechanical bonds, formed by the catenating ligand loops, that hold together the two [Pd**L**_2_] subunits and shape the cavity. To the best of our knowledge, this motif has never been reported before,^[28]^ while the multiple loop entanglement resembles seminal work by Böhmer and co‐workers on fully organic, tethered cavitands and Fujita *et al*. on a circular tris[2]catenane formed from three Pd‐based lemniscates.^[29]^

**Figure 4:**
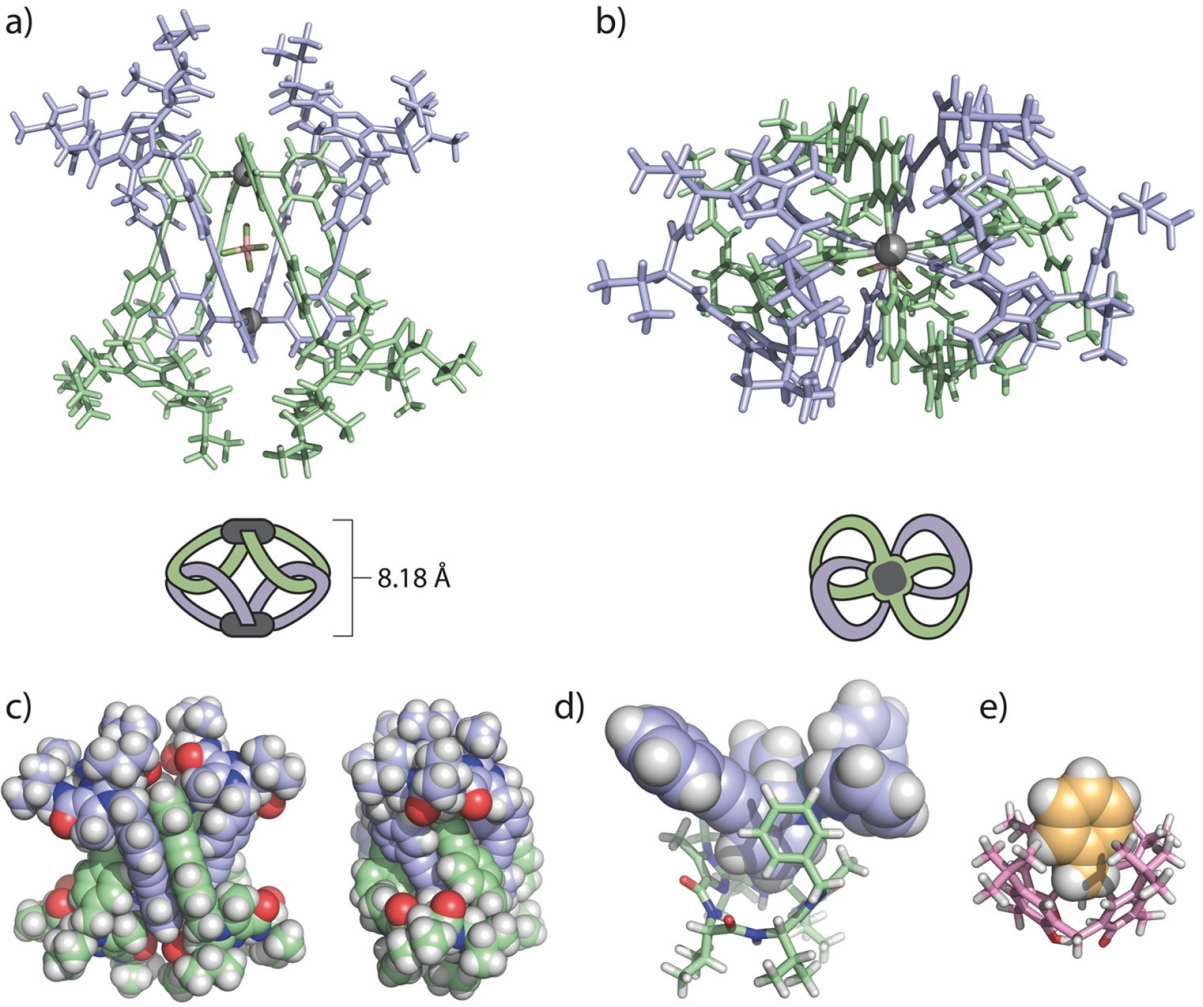
X‐ray structure of [BF_4_@Pd_2_ **L**_4_](BF_4_) (solvents and outer anions omitted for clarity): a) side and b) top view of the catenated motif; c) space filling side views; d) detail showing the non‐covalent nesting of one of the coordinating pyridines in the basked‐shaped macrocyclic backbone of another ligand and e) X‐ray structure of toluene@calix‐4‐arene for comparison.^[32]^

Closer examination of the structure reveals three features that should favour the formation of the interlocked dimer from an enthalpic point of view: 1) sandwiching of the BF_4_^−^ anion between the two metal centers, 2) π‐stacking between pairs of ligand arms (Figure 4 c) and—most intriguingly—3) embedding of four of the coordinating pyridines (two on either side) in the basket‐shaped cavities of the four ligands’ macrocyclic backbones (Figure 4 d). While chemically quite different, it is interesting to qualitatively compare this situation to the iconic dispersion‐driven calix‐4‐arene complex with toluene shown in Figure 4 e.

When revisiting the NMR spectra with the X‐ray structure in mind, the twofold splitting of all signals becomes understandable from a symmetry point of view, with each ligand featuring two distinguishable halves. NOESY contacts were found to be in well agreement with the crystal structure, as highlighted by contacts between protons H_a_/_a′_ to proton H_e_ (Figure 2 d) as well as between H_a_ to H_a′_ and H_b_ to H_b′_ which are only observed for the catenated assembly but not for the ligand itself. Signals belonging to the minor product largely resemble the peak pattern of the major species in terms of shifting and splitting effects. We therefore propose that the minor product is a structurally closely related interlocked isomer with lemniscate subunits showing an opposite sense of entanglement. A DFT‐based comparison between the isomers supports this hypothesis (Supporting Information).

Furthermore, a series of DFT calculations was conducted to gain insight into the enthalpic drive behind formation of this unusual type of catenane.^[30]^ While the sandwich configuration of the encapsulated anion between the two Pd^II^ centers was identified to contribute significant stabilization as compared to the bare pair of di‐cationic moieties arranged in 8.2 Å distance, we found further stabilization arising from π‐stacking interactions between the phenyl‐alkynyl‐pyridyl arms in pairs of ligands belonging to different palladium centers (Figure 4 c). Furthermore, embedding of the inner [Pd(phenyl‐alkynyl‐pyridine)_4_]_2_ core into a shell consisting of the four surrounding peptidic macrocycles was identified as stabilizing factor (Supporting Information). Hence, the whole system may be characterized to feature two different host‐guest interactions, a Coulomb‐driven anion encapsulation and a London dispersion‐driven nesting of strongly polarized aromatic residues inside macrocyclic hosts. DFT gas‐phase computations on ωB97X‐D/def2‐TZVP level of theory delivered for the latter effect a stabilizing contribution of 163 kJ mol^−1^ per corner (comparing to a stabilization of 85 kJ mol^−1^ for the non‐covalent toluene@calix‐4‐arene complex, computed under the same conditions).

In conclusion, a new structural variation of the well‐known class of [Pd_2_ **L**_4_] coordination cages was synthesized based on a chiral peptidic backbone. The interlocked topology creates a cavity between the two palladium cations, able to encapsulate one anion such as BF_4_^−^ or Cl^−^. The herein revealed principle of joining neighboring metal nodes not by direct ligand bridges but by mechanical bonds may be transferred to discrete structures of even higher nuclearity or extended networks. Furthermore, mechanical bonding has the potential to bestow metallo‐supramolecular assemblies with adaptive and stimuli‐responsive behavior, applicable in the context of allosteric receptors, adjustable catalytic confinements and soft materials.

## Experimental Section

Assemblies were formed upon addition of [Pd(CH_3_CN)_4_](X)_2_ (X=BF_4_/NTf_2_) (0.5 equiv.) to ligand **L** (1 equiv.) in CD_3_CN in the presence of Cl^−^ followed by heating at 70 °C overnight. Single crystals suitable for X‐ray crystal structure determination were grown by slow diffusion of ether into a mixture of **L** and [Pd(CH_3_CN)_4_](BF_4_)_2_ in CD_3_CN.

## Conflict of interest

The authors declare no conflict of interest.

## Supporting information

As a service to our authors and readers, this journal provides supporting information supplied by the authors. Such materials are peer reviewed and may be re‐organized for online delivery, but are not copy‐edited or typeset. Technical support issues arising from supporting information (other than missing files) should be addressed to the authors.

Supplementary
