# Supplementary material for: A New Mechanically‐Interlocked [Pd2L4] Cage Motif by Dimerization of two Peptide‐based Lemniscates
Source: Angew Chem Int Ed Engl. 2020 Oct 13;59(50):22489–93. doi: 10.1002/anie.202010995 (PMC7756597; doi:10.1002/anie.202010995)
Supplement: Supplementary file 1 — Supplementary [file ANIE-59-22489-s001.pdf]

Angewandte Chemie
Eine Zeitschrift der Gesellschaft Deutscher Chemiker

Supporting Information

A New Mechanically-Interlocked [Pd_2L_4] Cage Motif by Dimerization of two Peptide-based Lemniscates

*Thorben R. Schulte, Julian J. Holstein, Laura Schneider, Abdulselam Adam, Gebhard Haberhauer, and Guido H. Clever**

anie_202010995_sm_miscellaneous_information.pdf

1 General **1**

1.1 Synthesis of the compounds 2

1.1.1 Bidentate ligand L 2

1.1.1 [Cl@Pd_2L_4] 7

1.1.2 Mononuclear assembly [PdL_2] 11

2 Single-crystal X-ray Crystallography **12**

2.1 Crystal structure of [BF_4@Pd_2L_4]$^{3+}$ 12

Specific refinement details **13**

Table S1. Crystal data and structure refinement for [BF_4@Pd_2L_4]$^{3+}$ 14

3 Computational studies **15**

3.1 Comparison of plausible isomers 15

3.2 Dissection of contributions driving dimer formation 17

4 Ion Mobility Measurements **21**

5 References **22**

1 General

Where necessary, experiments were performed under nitrogen atmosphere using standard Schlenk techniques. Commercially available reagents were used as received without further purification, if not noted differently.

NMR measurements were conducted on Bruker Avance 500 III HD and Avance HD 600 spectrometers and an INOVA 600 MHz instrument from Varian. All chemical shifts (δ) are given in ppm. The spectra were referenced to the peak for the protium impurity in the deuterated solvents indicated in brackets in the analytical data. Mass spectrometric measurements were performed on timsTOF, Compact and BioTOF III ESI-TOF machines from Bruker.

1.1 Synthesis of the compounds

1.1.1 Bidentate ligand L

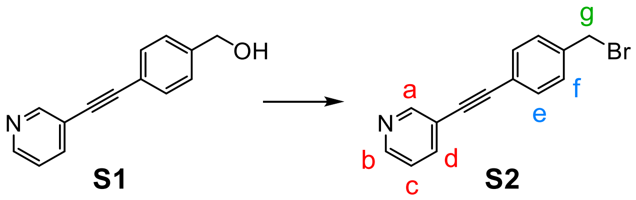

Bromide S2. Triphenylphosphine (513 mg, 1.96 mmol, 1.2 eq.) was dissolved under argon in DCM (5.6 mL) and cooled to 0 °C. Br_2 (300 mg, 1.87 mmol, 1.2 eq.) dissolved in DCM and benzyl alcohol **S1**[1] (341 mg, 1.63 mmol, 1.0 eq.) dissolved in DCM were successively and slowly added at 0 °C. The solution was stirred for one hour. Then DCM and *n*-pentane were added. The precipitated white solid was filtered off, washed with *n*-pentane and dried. The crude product was redissolved in DCM and mixed with *n*-pentane. The precipitated white solid was filtered off, washed with *n*-pentane and dried to yield **S2** (444 mg, 1.63 mmol, quant.) as a white solid.

^{1}H NMR (600 MHz, $CDCl_3$) δ = 4.50 (s, 2H, H_g), 7.46 (d, *J* = 8.3 Hz, 2H, H_f), 7.56 (d, *J* = 8.3 Hz, 2H, H_e), 7.99 (dd, *J* = 8.1, 5.8 Hz, 1H, H_c), 8.50 (dt, *J* = 8.1, 1.6 Hz, 1H, H_d), 8.79 (d, *J* = 5.4 Hz, 1H, H_b), 8.93 (d, *J* = 1.3 Hz, 1H, H_a).

^{13}C NMR (151 MHz, $CDCl_3$): δ = 32.22 (s; CH_2C_{ar}), 81.93 (q; C≡C), 98.61 (q; C≡C), 120.26 (q; C_{ar}), 124.83 (q; C_{ar}), 126.77 (t; C_{ar}), 129.44 (t; C_{ar}), 132.54 (t; C_{ar}), 138.77 (t; C_{ar}), 140.33 (q; C_{ar}), 142.74 (t; C_{ar}), 147.31 ppm (t; C_{ar}).

HRMS (ESI): $[C_{14}H_{10}{}^{79}BrN+H]^+$: calculated: 272.0069; observed: 272.0084.

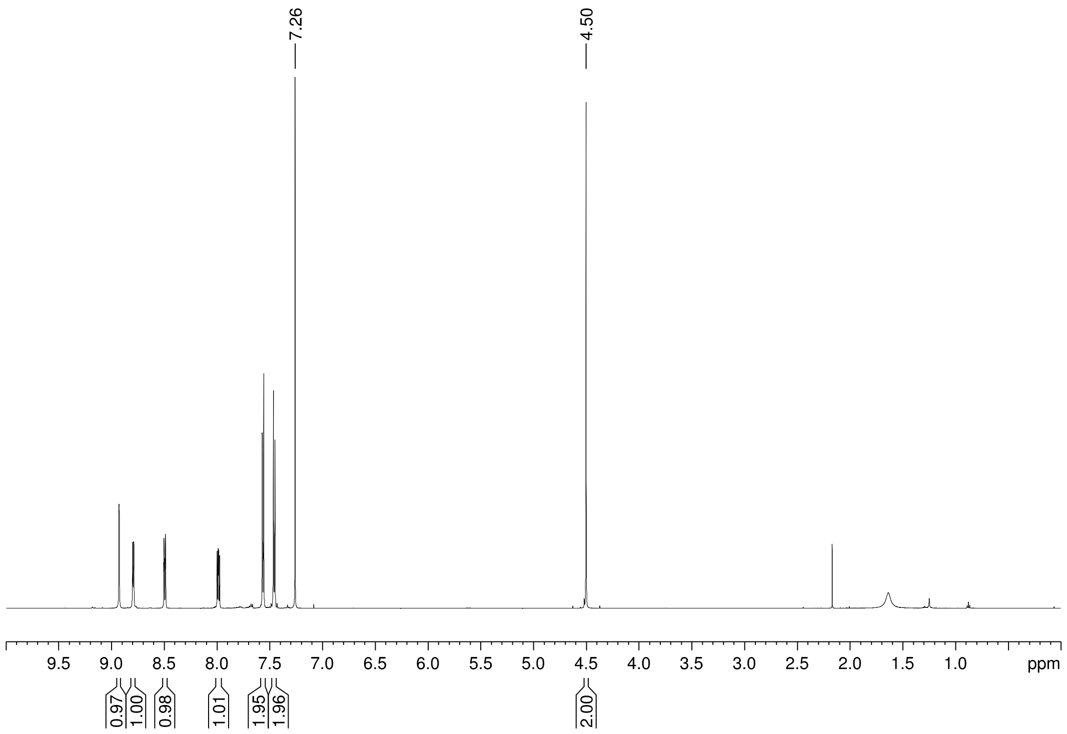

Figure S1. ^{1}H NMR spectrum of benzyl bromide **S2** (600 MHz) in $CDCl_3$.

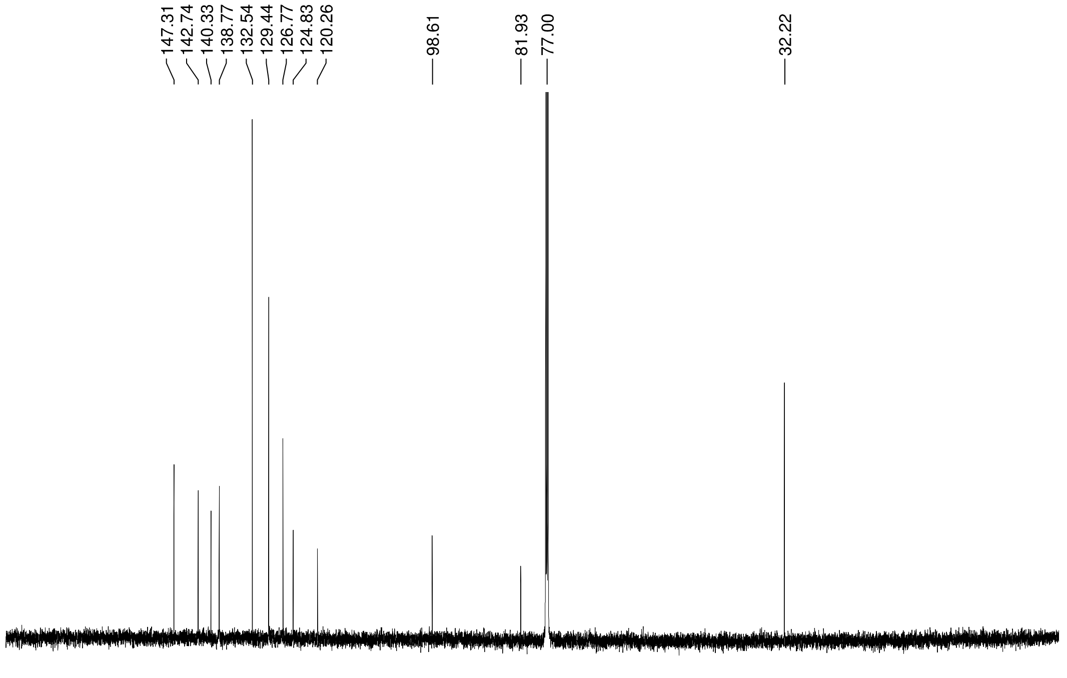

Figure S2. ^{13}C NMR spectrum of benzyl bromide **S2** (151 MHz) in $CDCl_3$.

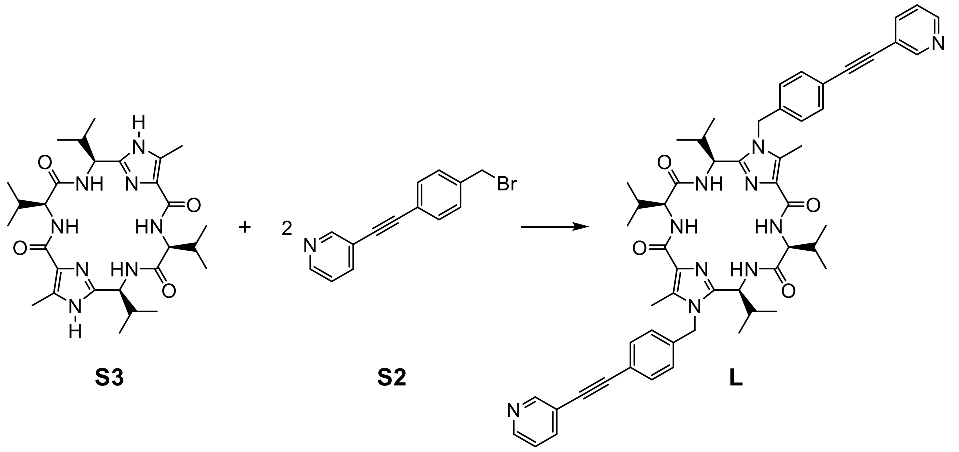

Ligand L. To a solution of cyclic peptide **S3**[2] (30 mg, 0.054 mmol, 1.0 eq.) in acetonitrile (20 mL), cesium carbonate (176 mg, 0.539 mmol, 10 eq.) was added under an argon atmosphere at room temperature. Subsequently, benzyl bromide **S2** (59 mg, 0.22 mmol, 4.0 eq.) was added in portions and the solution was stirred overnight. The solvent was evaporated to dryness, the residue was dissolved in DCM and washed with water. The aqueous layer was saturated with NaCl and then repeatedly extracted with DCM. The organic layers were combined, dried over $MgSO_4$ and concentrated in vacuo. The residue was purified by flash column chromatography with silica gel (DCM/MeOH 94:6) to yield ligand **L** (32 mg, 0.034 mmol, 63 %) as a white solid.

^{1}H NMR (600 MHz, $CDCl_3$) δ = 0.63 (d, *J* = 6.6 Hz, 6H, $H_{l/l'}$), 0.97 (d, *J* = 6.7 Hz, 6H, $H_{l/l'}$), 1.05 (d, *J* = 6.7 Hz, 6H, $H_{l/l'}$), 1.08 (d, *J* = 6.8 Hz, 6H, $H_{l/l'}$), 2.18-2.28 (m, 4H, $H_{k+k'}$), 2.43 (s, 6H, H_h), 4.45 (dd, *J* = 9.2, 6.9 Hz, 2H, H_j), 4.76 (t, *J* = 9.0 Hz, 2H, $H_{j'}$), 5.09 (d, *J* = 17.2 Hz, 2H, H_g), 5.26 (d, *J* = 17.4 Hz, 2H, H_g), 6.75 (d, *J* = 8.5 Hz, 2H, $H_{i'}$), 6.98 (d, *J* = 8.1 Hz, 4H, H_f), 7.02 (d, *J* = 9.2 Hz, 2H, H_i), 7.25-7.28 (m, 2H, H_c), 7.50 (d, *J* = 8.2 Hz, 4H, H_e), 7.77-7.94 (m, 2H, H_d), 8.53 (s, 2H, H_b), 8.74 (s, 2H, H_a). Color coding see Fig. S6. Protons H_l, $H_{l'}$ and H_g give rise to diastereotopic splitting, however, unambiguous assignment was not possible.

^{13}C NMR (151 MHz, $CDCl_3$): δ = 9.77 (p; $C_{imi}\mathit{C}H_3$), 18.53 (p; $CH(\mathit{C}H_3)_2$), 19.04 (p; $CH(\mathit{C}H_3)_2$), 19.39 (p; $CH(\mathit{C}H_3)_2$), 19.43 (p; $CH(\mathit{C}H_3)_2$), 30.47 (t; $\mathit{C}H(CH_3)_2$), 32.53 (t; $\mathit{C}H(CH_3)_2$), 46.68 (s, $\mathit{C}H_2C_{ar}$), 50.77 (t; CHNH), 59.60 (t; CHNH), 86.54 (q; C≡C), 91.86 (q; C≡C), 120.17 (q; C_{py}), 122.38 (q; C_{ar}), 122.99 (t; C_{py}), 126.27 (t; C_{ar}), 129.59 (q; C_{imi}), 132.30 (t; C_{ar}), 133.18 (q; C_{imi}), 135.97 (q; C_{ar}), 138.40 (t; C_{py}), 147.88 (q; C_{imi}), 148.60 (t; C_{py}), 152.16 (t; C_{py}), 162.89 (CO), 171.19 ppm (CO).

HRMS (ESI): $[C_{56}H_{62}N_{10}O_4+H]^+$: calculated: 939.5028; observed: 939.5003.

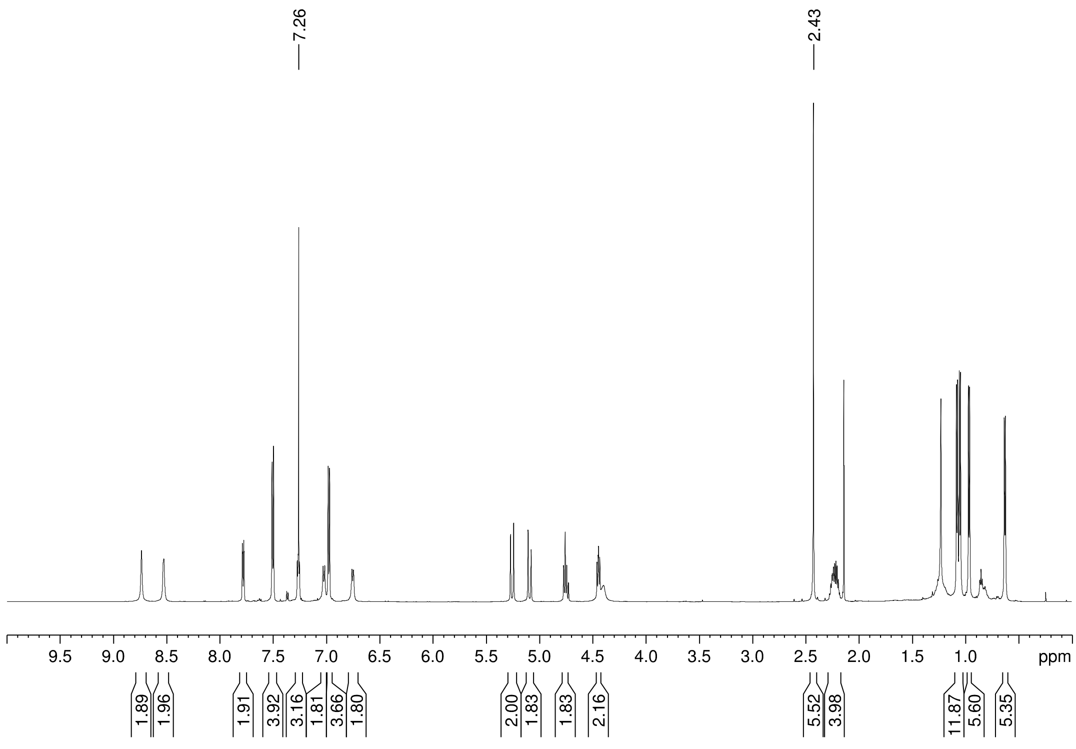

Figure S3. ^{1}H NMR spectrum of ligand **L** (600 MHz) in $CDCl_3$.

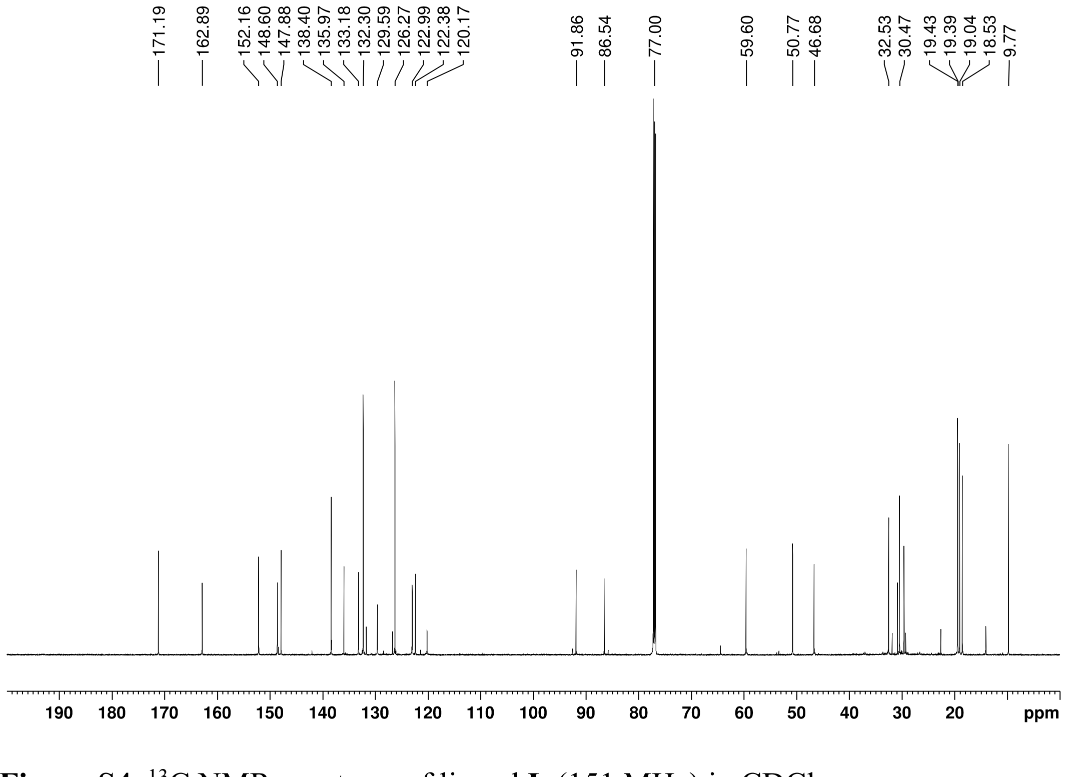

Figure S4. ^{13}C NMR spectrum of ligand **L** (151 MHz) in $CDCl_3$.

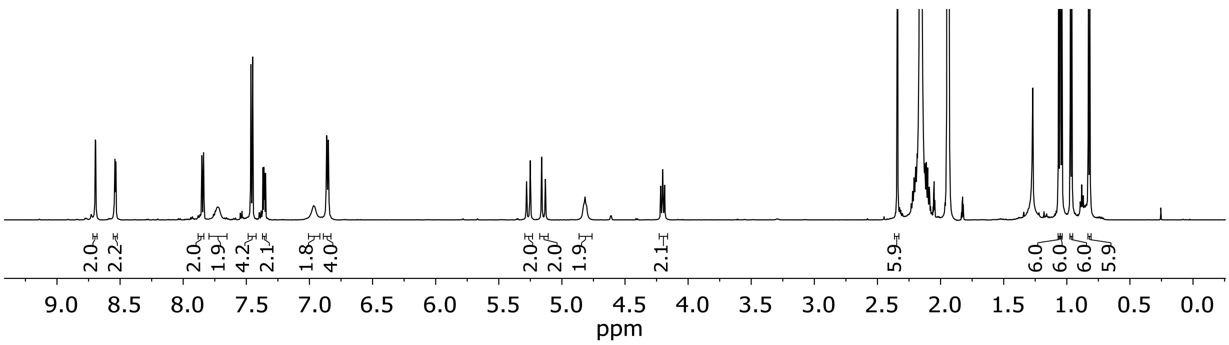

Figure S5. ^{1}H NMR spectrum of **L** (500 MHz) in CD_3CN.

^{1}H NMR (500 MHz, CD_3CN) δ = 0.82 (d, *J* = 6.8 Hz, 6H, $H_{l/l'}$), 0.97 (d, *J* = 6.8 Hz, 6H, $H_{l/l'}$), 1.04 (d, *J* = 6.8 Hz, 6H, $H_{l/l'}$), 1.06 (d, *J* = 6.8 Hz, 6H, $H_{l/l'}$), 2.16 (m, 4H, partially overlapped by water signal, $H_{k+k'}$), 2.34 (s, 6H, H_h), 4.20 (t, *J* = 9.3 Hz, 2H, H_j), 4.82 (t, *J* = 6.4 Hz, 2H, $H_{j'}$), 5.15 (d, *J* = 17.5 Hz, 2H, H_g), 5.27 (d, *J* = 17.5 Hz, 2H, H_g), 6.86 (d, *J* = 8.0 Hz, 4H, H_f), 6.96 (s, 2H, $H_{i'}$), 7.36 (ddd, *J* = 8.0, 4.9, 0.9 Hz, 2H, H_c), 7.46 (d, *J* = 8.3 Hz, 4H, H_e), 7.74 (d, *J* = 12.0 Hz, 2H, H_i), 7.85 (dt, *J* = 7.9, 1.9 Hz, 2H, H_d), 8.54 (dd, *J* = 4.8, 1.7 Hz, 2H, H_b), 8.69 (d, *J* = 1.3 Hz, 2H, H_a). Color coding see Fig. S6. Protons H_l, $H_{l'}$ and H_g give rise to diastereotopic splitting, however, unambiguous assignment was not possible.

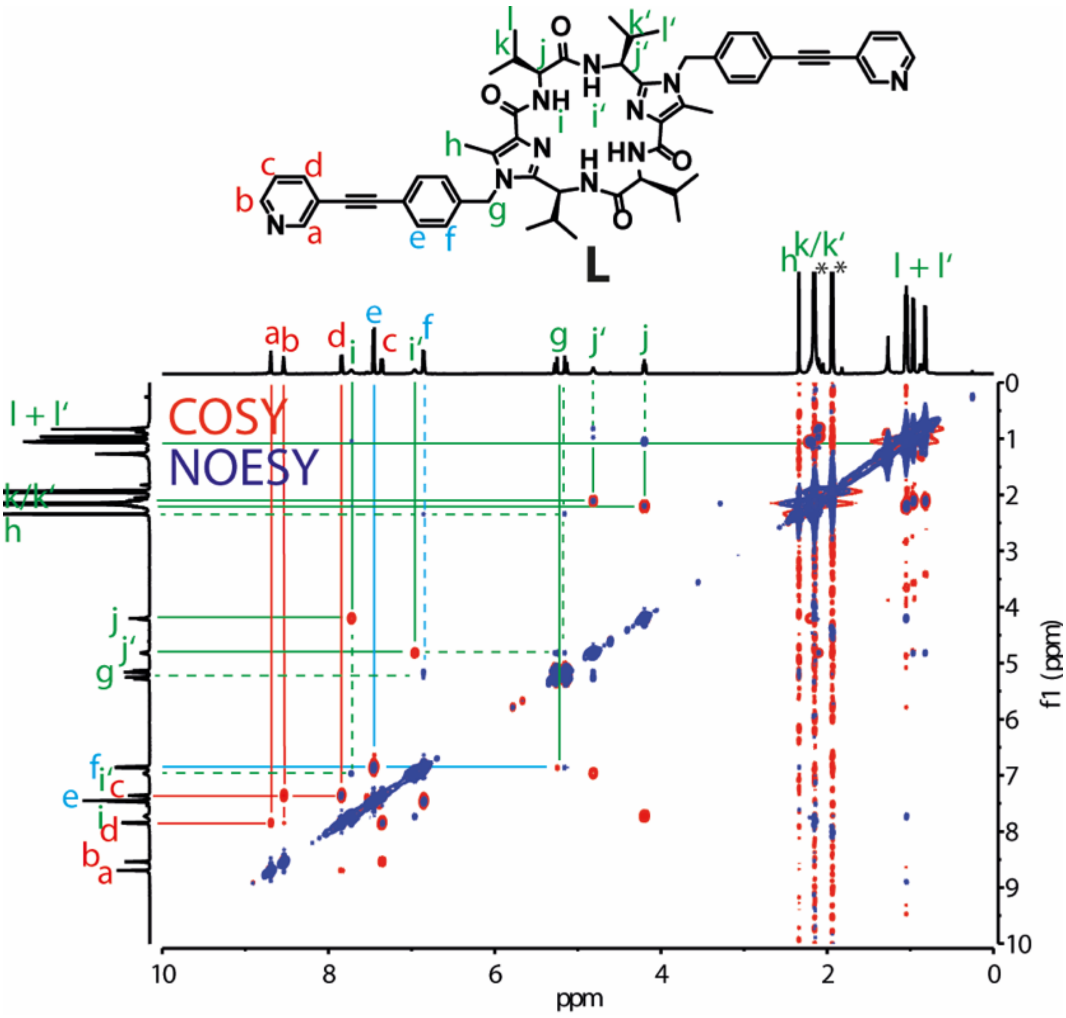

Figure S6. COSY (red) and NOESY (blue) spectra of **L** (500 MHz) in CD_3CN superimposed. COSY contacts are marked with solid lines and NOESY contacts with dashed lines.

1.1.1 [Cl@Pd_2L_4]

[Cl@Pd_2**L**$_4$]$(X)_3$ assemblies were prepared in quantitative yields by mixing ligand **L** (1.3 mg, 1.4 µmol, 2.0 eq.) in 550 µL CD_3CN with 57 µL of a 15 mM CD_3CN solution of $[Pd(CH_3N)_4](BF_4)_2$ (for X = BF_4^-; 369 µg, 0.83 µmol, 1.2 eq.) and stirring at r.t. for 2h. A 36 mM CD_3CN solution of NBu_4Cl (125 µg, 0.46 µmol, 0.65 eq) was then added stepwise until vanishing of the NMR signal at 4.1 ppm (dashed red lines in figure S7c), as monitored via ^1H NMR spectroscopy. The mixture was stirred over night at 70 °C leading to the target structure as the main product (Figure S7d).

For X = PF_6^-, SbF_6^- and NTf_2^-, $[Pd(CH_3CN)_4](PF_6)_2$, $[Pd(CH_3CN)_4](SbF_6)_2$ and $[Pd(CH_3CN)_4](NTf_2)_2$ were prepared in situ by reacting $[Pd(CH_3CN)_2Cl_2]$ with the respective silver salt in a 1:2 ratio for 30 min and decanting the solution. In case of these Pd sources,

addition of NBu_4Cl was not found necessary to prepare [Cl@Pd_2**L**$_4$](X)$_3$ due to the presence of residual chloride in the solutions, leading in all cases to basically the same ^{1}H NMR spectra as with X = BF_4^- after titration with NBu_4Cl.

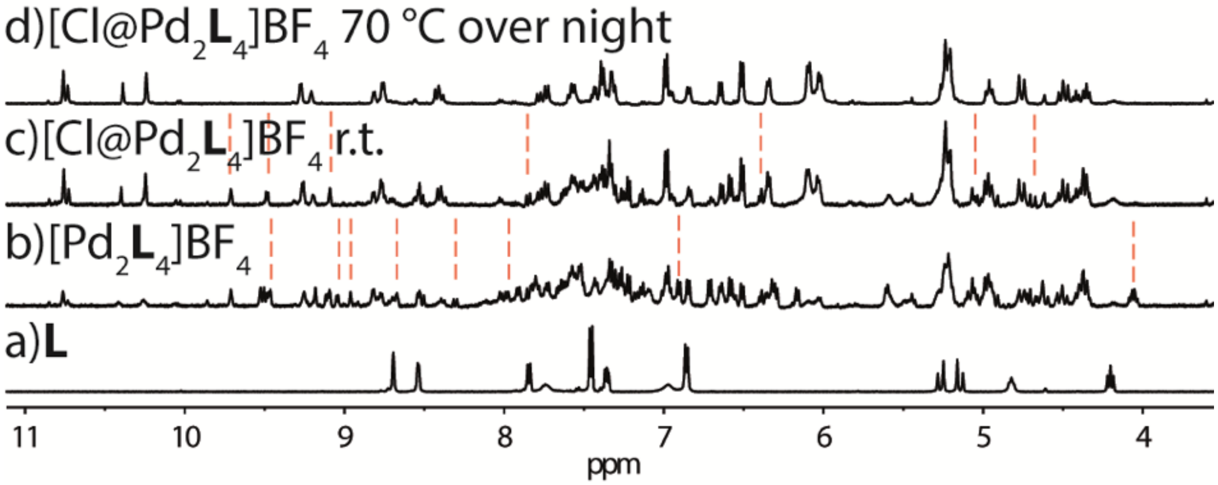

Figure S7. ^{1}H NMR spectra in CD_3CN (500 MHz) showing different steps in the formation of target compound [Cl@Pd_2**L**$_4$](X)$_3$: a) ligand L; b) after addition of 0.5 eq. [Pd(CH_3CN)$_4$](BF_4)$_2$; c) after addition of 1 eq. NBu_4Cl at r.t. (red dotted lines indicate vanishing signals); d) after heating over night at 70 °C.

Besides the major isomer (assigned to the topology as observed in the X-ray structure), all samples contained varying amounts (20 - 30%) of a minor isomer (see computational section for a plausible structure), also featuring NMR signal splitting into two sets, in part overlapping with the signals of the major species.

Several signals of the major species that were found to overlap with the minor component, the NBu_4^+ counter cation and solvent signals in the 1D spectrum could be assigned nevertheless with help of 2D spectra (Figure S9).

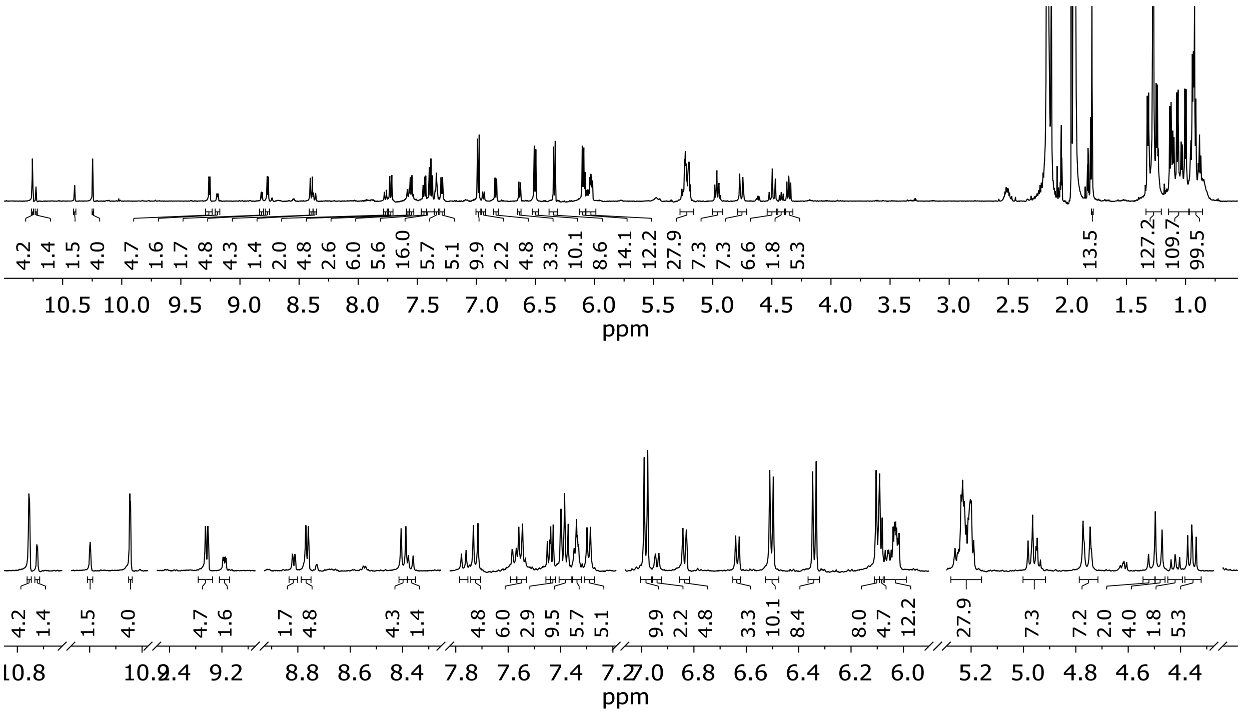

Figure S8. ^{1}H NMR of [Cl@Pd$_2$**L**$_4$]. Top: full spectrum, bottom: enlarged detail.

The following listing contains all observed signals (including patterns of overlapped signals) above 4 ppm (labels containing * stand for all diastereotopic signals associated with protons carrying the same letter). Signals of the major species that could be assigned are referenced by their proton label and color code (Fig. S9). Estimated contributions to the observed total integrals of overlapping signals are given where appropriate.

^{1}**H NMR** (600 MHz, CD$_3$CN) δ = 4.36 (dd, *J* = 10.4, 9.1 Hz, 5H, 4H$_{j*}$), 4.40 – 4.44 (m, 1.8H), 4.48 (d, *J* = 15.5 Hz, 4H, 4H$_{g*}$), 4.51 (d, *J* = 15.5 Hz, 2H), 4.76 (d, *J* = 16.0 Hz, 7.2H, 4H$_{g*}$), 4.96 (d, J = 15.5 Hz, 7.3H, 4H$_{j*}$), 5.17 – 5.28 (m, 27.9H, 8H$_{j*}$ + 8H$_{g*}$), 6.00 – 6.05 (m, 12H, 4H$_{c'}$), 6.09 (d, *J* = 7.9 Hz, 4.7H), 6.10 (d, *J* = 7.8 Hz, 8H, 8H$_{f}$), 6.34 (d, *J* = 8.1 Hz, 8H, 8H$_{e}$), 6.50 (d, *J* = 8.0 Hz, 10H, 8H$_{f'}$), 6.63 (d, *J* = 8.0 Hz, 3.3H), 6.84 (dt, *J* = 8.0, 1.6 Hz, 4.8H, 4H$_{d'}$), 6.94 (d, *J* = 8.0 Hz, 2.2H), 6.98 (d, *J* = 8.1 Hz, 10H, 8H$_{e'}$), 7.29 (d, *J* = 7.8 Hz, 5H, 4H$_{i*}$), 7.34 (q, *J* = 3.1, 2.4 Hz, 5.7H), 7.37 – 7.40 (m, 9.5H, 4H$_{c}$ + 4H$_{d}$), 7.43 (d, *J* = 5.7 Hz, 2.9H), 7.45 (d, *J* = 5.8 Hz, 2.4H), 7.55 (d, *J* = 8.5 Hz, 6H, 4H$_{i*}$), 7.55 – 7.60 (m, 2.6H), 7.72 (d, *J* = 10.5 Hz, 4.8H, 4H$_{i*}$), 7.77 (d, *J* = 10.7 Hz, 2H), 8.37 (d, *J* = 10.8 Hz, 1.4H), 8.40 (d, *J* = 10.8 Hz, 4H, 4H$_{i*}$), 8.76 (dd, *J* = 5.9, 1.4 Hz, 4H, 4H$_{b'}$), 8.82 (s, 1.7H), 9.19 (dd, *J* = 4.9, 2.3 Hz), 9.26 (dd, *J* = 5.7, 1.4 Hz, 4H, 4H$_{b}$), 10.25 (d, *J* = 1.8 Hz, 4H, 4H$_{a}$), 10.40 (s), 10.72 (d, *J* = 1.8 Hz), 10.75 (d, *J* = 1.8 Hz, 4H, 4H$_{a'}$).

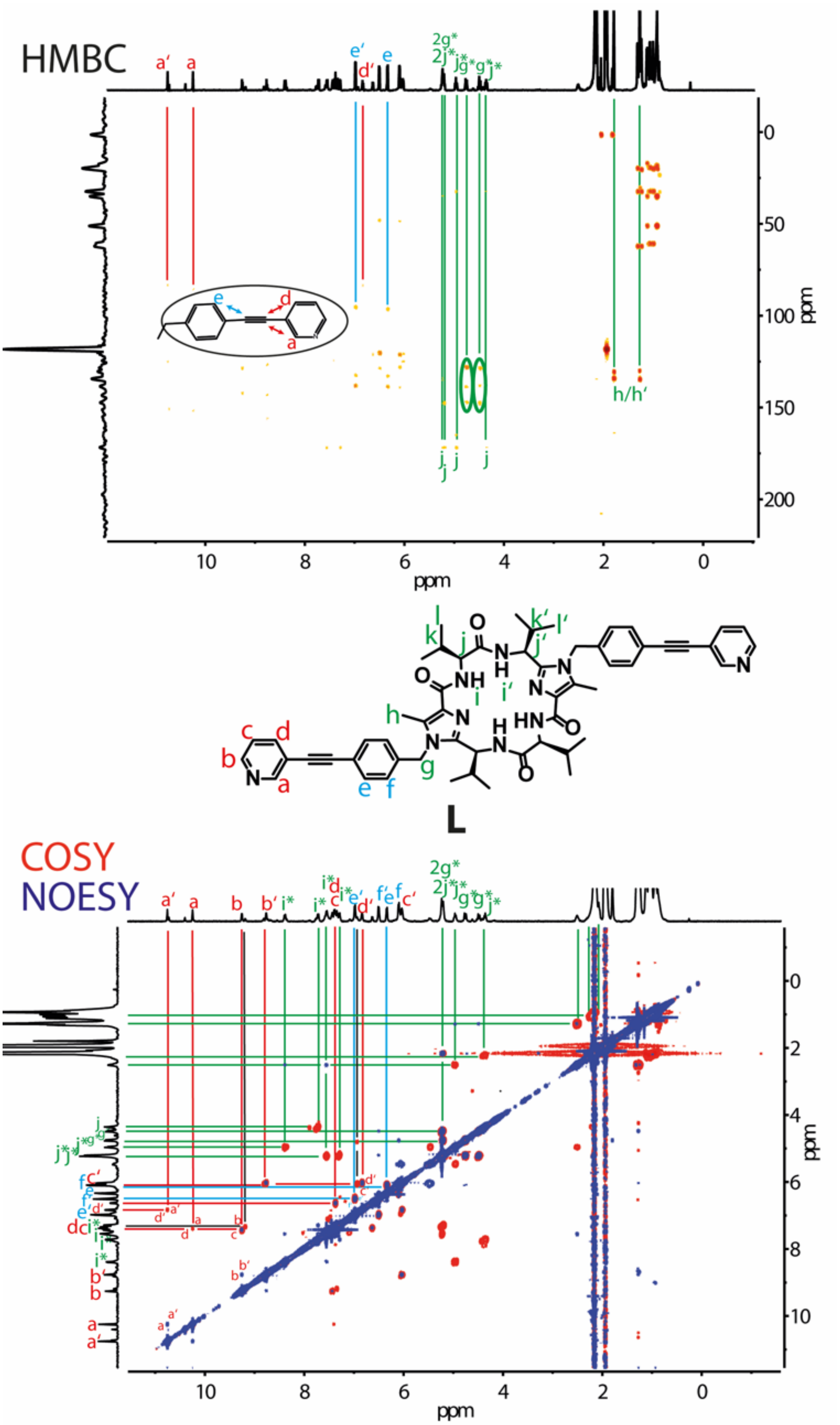

Figure S9. HMBC (600 MHz), combined COSY (red) and NOESY (blue) (both 500 MHz) spectra of $[Cl@Pd_2\mathbf{L}_4]^{4+}$ with NTf_2^- as counter ion in CD_3CN. Contacts are marked with dashed lines for NOESY contacts and solid line for COSY contacts.

1.1.2 Mononuclear assembly [PdL_2]

Figure-eight-shaped assembly [Pd**L**$_2$] was prepared by mixing ligand **L** (1.3 mg, 0.86 µmol, 2.0 eq.) in 550 µL DMSO-d6 with 30 µL of a 15 mM DMSO-d6 solution of [Pd(CH_3N)$_4$](BF_4)$_2$ (221 µg, 0.50 µmol, 1.2 eq.) and stirring at r.t. for 2h.

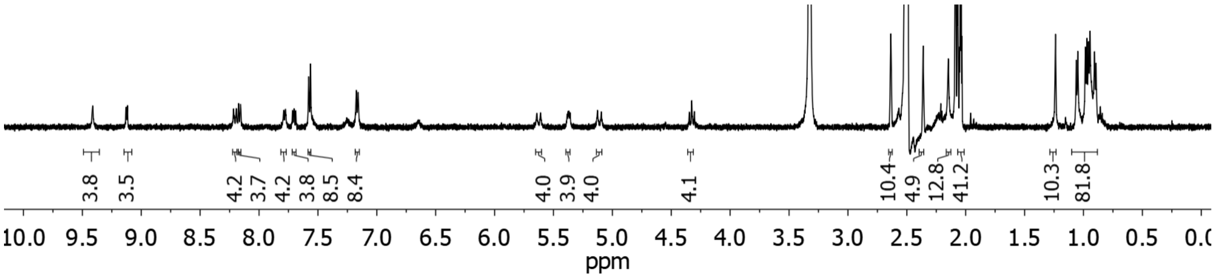

Figure S10. ^{1}H NMR of [Pd**L**$_2$](BF_4)$_2$ in DMSO-d6.

^{1}H NMR (500 MHz, DMSO-d_6) δ = 4.33 (t, J = 10.8 Hz, 4H, H_{j*}), 5.11 (d, J = 16.1 Hz, 4H, H_{g*}), 5.34 – 5.42 (m, 4H, H_{j*}), 5.63 (d, J = 16.3 Hz, 4H, H_{g*}), 7.17 (d, J = 7.9 Hz, 8H, H_f), 7.57 (d, J = 7.9 Hz, 8H, H_e), 7.70 (dd, J = 7.8, 6.0 Hz, 4H, H_c), 7.78 (d, J = 8.2 Hz, 4H, H_d), 8.17 (d, J = 8.3 Hz, 4H, H_{i*}), 8.20 (d, J = 10.8 Hz, 4H, H_{i*}), 9.12 (d, J = 5.7 Hz, 4H, H_b), 9.41 (s, 4H, H_a).

Mass analysis: see main manuscript.

2 Single-crystal X-ray Crystallography

2.1 Crystal structure of $[BF_4@Pd_2\mathbf{L}_4]^{3+}$

Single crystals suitable for X-ray diffraction were obtained by diffusion of Et_2O into a solution of $[BF_4@Pd_2\mathbf{L}_4]^{3+}$ in acetonitrile. Single crystals of $[BF_4@Pd_2\mathbf{L}_4]^{3+}$ were transferred onto a glass slide covered by NVH oil. Four crystals were quickly mounted onto 200 µm nylon loops and immediately flash cooled in liquid nitrogen to avoid collapse of the crystal lattice.

Crystals were stored at cryogenic temperature in dry shippers, in which they were safely transported to macromolecular beamline P_{11},[3] PETRA III, DESY, Hamburg, Germany. Samples were mounted using the StäubliTX60L robotic arm. A wavelength of $\lambda = 0.6889$ Å was chosen using a liquid N_2 cooled double crystal monochromator. Single crystal X-ray diffraction data was collected at 80(2) K on a single axis goniometer, equipped with an Oxford Cryostream 800 and a Pilatus 6M fast. 1800 diffraction images were collected in a 360° φ sweep at a detector distance of 200 mm, 100% filter transmission, 0.2° step width and 0.2 second exposure time per image. Integration was done with XDS.[4]

The data was cut at 1.1 Å, as the signal to noise ratio has dropped below $I/\sigma(I) < 4.0$. Due to high mosaicity and disorder in the solvent region a higher resolution could not be achieved. Nevertheless, the resolution achieved was sufficient to solve the structure by intrinsic phasing/direct methods using SHELXT.[5] SHELXL[6] (version 2014/7) was used for refinement and ShelXle7 as a graphical user interface. The DSR program plugin was employed for modelling.[8-9] All cycles were refined against F^2 until convergence using the conjugate-gradient algorithm (CGLS). Only for computing the crystallographic information file (CIF) the full-matrix least-squares routine was employed. All non-hydrogen atoms were refined with anisotropic displacement parameters. The hydrogen atoms were refined isotropically on calculated positions using a riding model with their U_{iso} values constrained to 1.5 times the U_{eq} of their pivot atoms for terminal sp^3 carbon atoms and 1.2 times for all other carbon atoms. Crystallographic data (including structure factors) for the structures reported in this paper have been deposited with the Cambridge Crystallographic Data Centre. CCDC 1976541 contain the supplementary crystallographic data for this paper. Copies of the data can be obtained free of charge from the Cambridge Crystallographic Data Centre via www.ccdc.cam.ac.uk/structures.

Specific refinement details

In order to generate a molecular model and increase robustness of the refinement we have adapted and exploited techniques commonly applied in macromolecular structure refinement. A theoretical model bases on MMFFS force field was used as input for stereochemical restraints generation by the GRADE program using the GRADE Web Server (http://grade.globalphasing.org). The restraint dictionary was applied to the ligand in the refinement using residue name HAP. GRADE dictionary for SHELXL contains target values and standard deviations for 1.2-distances (DFIX) and 1.3-distances (DANG), as well as restraints for planar groups (FLAT). The refinement of ADP's for non-hydrogen atoms was enabled by using the rigid bond restraint (RIGU)[10] in the SHELXL program in combination with SIMU restraints. The contribution of the electron density from disordered counterions, and solvent molecules, which could not be modelled with discrete atomic positions were handled using the SQUEEZE[11] routine in PLATON.[12] The solvent mask file (.fab) computed by PLATON was included in the SHELXL refinement via the ABIN instruction leaving the measured intensities untouched.

Table S1. Crystal data and structure refinement for $[BF_4@Pd_2L_4]^{3+}$

CCDC number	1976541
Sample code	tsp292
Empirical formula	$C_{224}H_{248}BF_4N_{40}O_{16}Pd_2$
Formula weight	4056.22
Temperature [K]	80(2)
Crystal system	monoclinic
Space group (number)	$P2_1$ (4)
a [Å]	20.074(4)
b [Å]	25.946(5)
c [Å]	52.329(11)
α [Å]	90
β [Å]	98.46(3)
γ [Å]	90
Volume [Å^3]	26958(10)
Z	4
ρ_{calc} [g/cm^3]	0.999
μ [mm^{-1}]	0.176
F(000)	8532
Crystal size [mm^3]	0.200×0.100×0.050
Crystal colour	colourless
Crystal shape	needle
Radiation	synchrotron (λ=0.6888 Å)
2Θ range [°]	1.53 to 36.49 (1.10 Å)
Index ranges	-18 ≤ h ≤ 18<br>-23 ≤ k ≤ 23<br>-47 ≤ l ≤ 47
Reflections collected	132862
Independent reflections	41875<br>R_{int} = 0.0683<br>R_{sigma} = 0.0651
Completeness to θ = 18.245°	99.6 %
Data / Restraints / Parameters	41875/10161/5169
Goodness-of-fit on F^2	1.721
Final R indexes [I≥2σ(I)]	R_1 = 0.1443<br>wR_2 = 0.3731
Final R indexes [all data]	R_1 = 0.1524<br>wR_2 = 0.3803
Largest peak/hole [eÅ^3]	1.14/-0.54
Flack X parameter	0.295(7)

3 Computational studies

3.1 Comparison of plausible isomers

Table S2. Relative energies of three isomeric species obeying the formula $[BF_4@Pd_2L_4]^{3+}$ were calculated by performing unconstrained geometry pre-optimizations on PM6 semiempiric level (Wavefunction Spartan '18)[13], then further optimization on B97-3c[14] level of theory in the ORCA software,[15] followed by single point energy calculations (DFT ωB97X-D/def2-SVP) in Wavefunction Spartan '18 (all gas phase). Calculations were performed for the interlocked topology as observed by single crystal X-ray structure determination and a diastereomeric form in which the ligand-related stereo centers retain the same configuration but the helical interlocking mode (giving rise to an additional mode of axial chirality) is opposite to the experimentally (X-ray) observed situation. The DFT-computed energies of both interlocked isomers were found to be in a similar range (within 11 kJ/mol for the gas phase models), suggesting that both isomers could be accessible in solution. While the X-ray structure-matching topology turned out to feature the higher energy, we denote this to the rather coarse theory level that had to be chosen due to the immense aggregate size (535 atoms) under negligence of solvation effects and further counter anions (also note that BF_4^--only containing sample showed a more complex NMR behavior than the Cl^--encapsulating species which, however, was not considered in the computational study). In accordance with 2D NMR data for the Cl^--encapsulating species, we therefore propose assignment of the major solution species to the diastereomer observed in the X-ray structure and the minor species revealed in the NMR results to the alternative interlocked topology.

Also, a non-interlocked $[BF_4@Pd_2L_4]^{3+}$ structure with all four ligands bridging both Pd cations in the classical lantern-shaped geometry was calculated. Although convergence issues were encountered during the geometry optimization, the latter structure turned out to be of significantly higher energy than the interlocked isomers. As such a non-interlocked structure could also be ruled out on the basis of the NMR splitting and NOESY data, the existence of a lantern-shaped Pd_2L_4 topology can be dismissed in the equilibrated reaction mixture.

isomer	rel. energy (kJ/mol)	B97-3c model
interlocked as in X-ray structure	B97-3c: 0<br>ωB97X-D/def2-SVP: 0	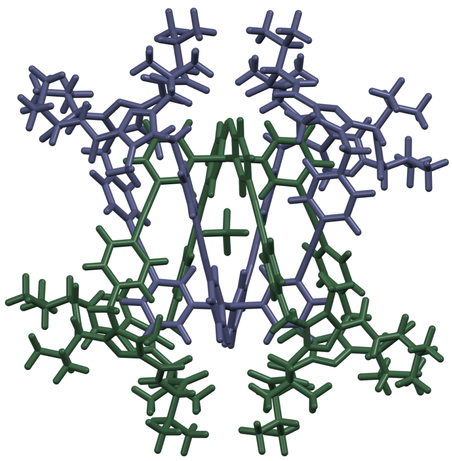
tentative interlocked diastereomer	B97-3c: - 11<br>ωB97X-D/def2-SVP: - 11	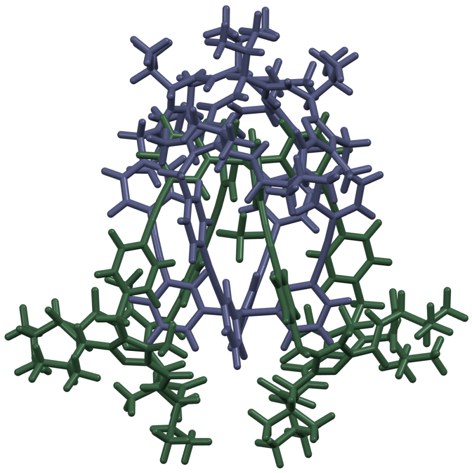
non-interlocked, lantern-shaped	B97-3c: + 373*<br>ωB97X-D/def2-SVP: n.d.	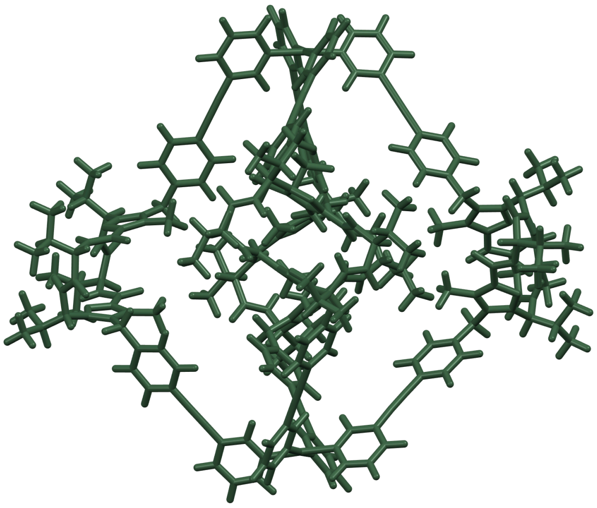

*geometry optimization terminated prematurely due to convergence issues

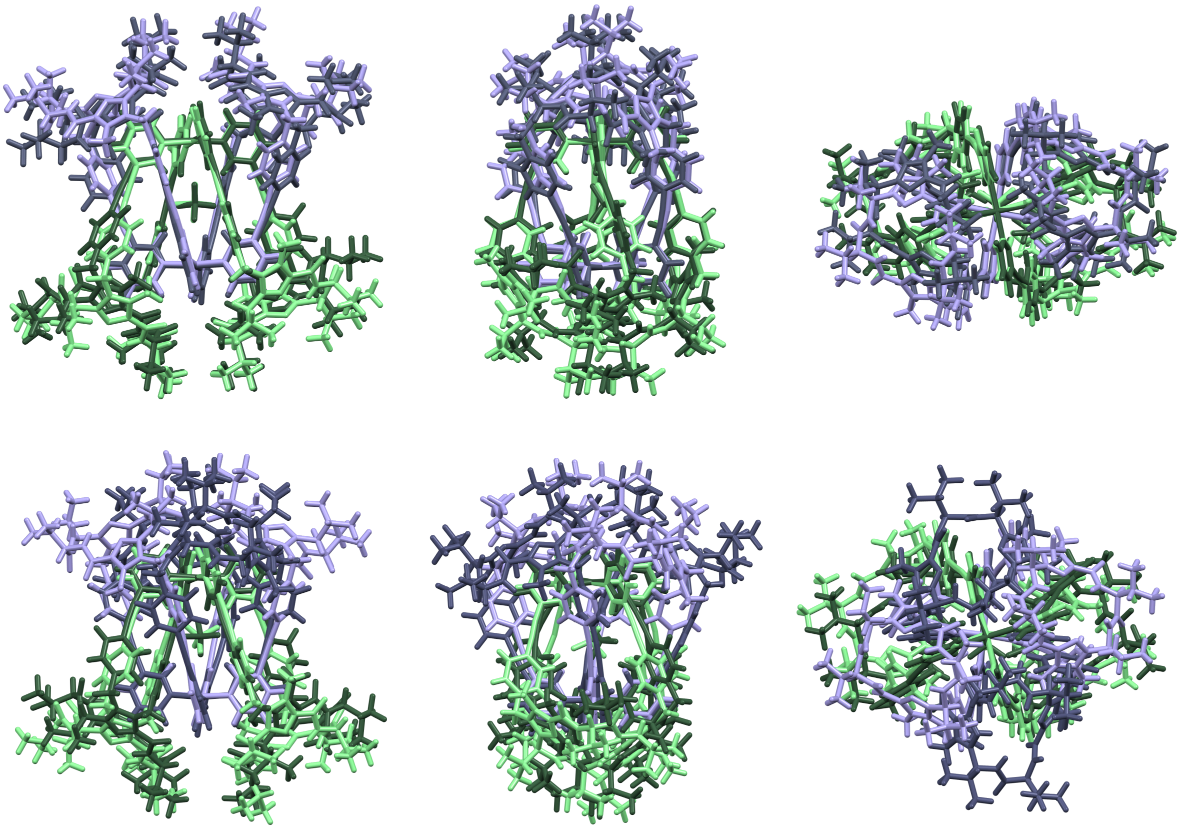

Figure S11. Three superimposed views, each, comparing the X-ray structure (light blue/green; Pd-Pd distance 8.18 Å) and B97-3c calculated structure (dark blue/green) of top: the interlocked topology as found in the crystal structure (Pd-Pd distance 8.30 Å) and bottom: the interlocked isomer (Pd-Pd distance 8.24 Å).
Left and middle: views perpendicular to Pd_2-axis, right: view along Pd_2-axis.

3.2 Dissection of contributions driving dimer formation

In order to get insight into the enthalpic contributions driving dimerization of the mononuclear lemniscates, a number of calculations were performed. Therefore, one of the almost identical $[BF_4@Pd_2L_4]^{3+}$ units found in the asymmetric unit of the X-ray structure including the encapsulated BF_4^- anion was chosen for computations carried out in the Spartan '18 software package.[13] First, all nuclear positions except those of the hydrogen atoms were frozen. The latter were then optimized on semiempiric PM6 level of theory to correct CH and XH bond lengths with respect to the ones generated throughout X-ray refinement. Based on this structure, DFT single point calculations on ωB97X-D/6-31G* (LanL2DZ for Pd(II)) and ωB97X-D/def2-TZVP levels of theory were performed for the entire $[BF_4@Pd_2L_4]^{3+}$ moiety and the following fragments, cut out from the whole assembly:

species	E(ωB97X-D/6-31G*-LanL2DZ)/Hartree	E(ωB97X-D/def2-TZVP)/Hartree
$[BF_4@Pd_2L_4]^{3+}$	-12752.631902	-12759.411024
tetra-cation $[Pd_2L_4]^{4+}$	-12327.878016	-12334.476430
free BF^{4-}	-424.375198	-424.596233
figure-eight-shaped half $[PdL_2]^{2+}$	-6163.852987	-6167.175087
fragment $\{[(Pd(3\text{-}phenylalkynylpyridine)_4]_2\}^{4+}$	-4694.954377	-4698.887584
half of this, $[(Pd(3\text{-}phenylalkynylpyridine)_4]^{2+}$	-2347.520448	-2349.496640
fragment $\{[(Pd(pyridine)_4]_2\}^{4+}$	-2238.033187	-2241.157141
half of this, $[Pd(pyridine)_4]^{2+}$	-1119.118640	-1120.681461
"outer shell" of four bis(phenyl-methylene)-substituted macrocycles	-9489.950304	-9493.292901
free benzene	-232.162076	-232.243978
For comparison:		
toluene@calix[4]arene	-	-2283.021324
free calix[4]arene	-	-2011.448676
free toluene	-	-271.540139

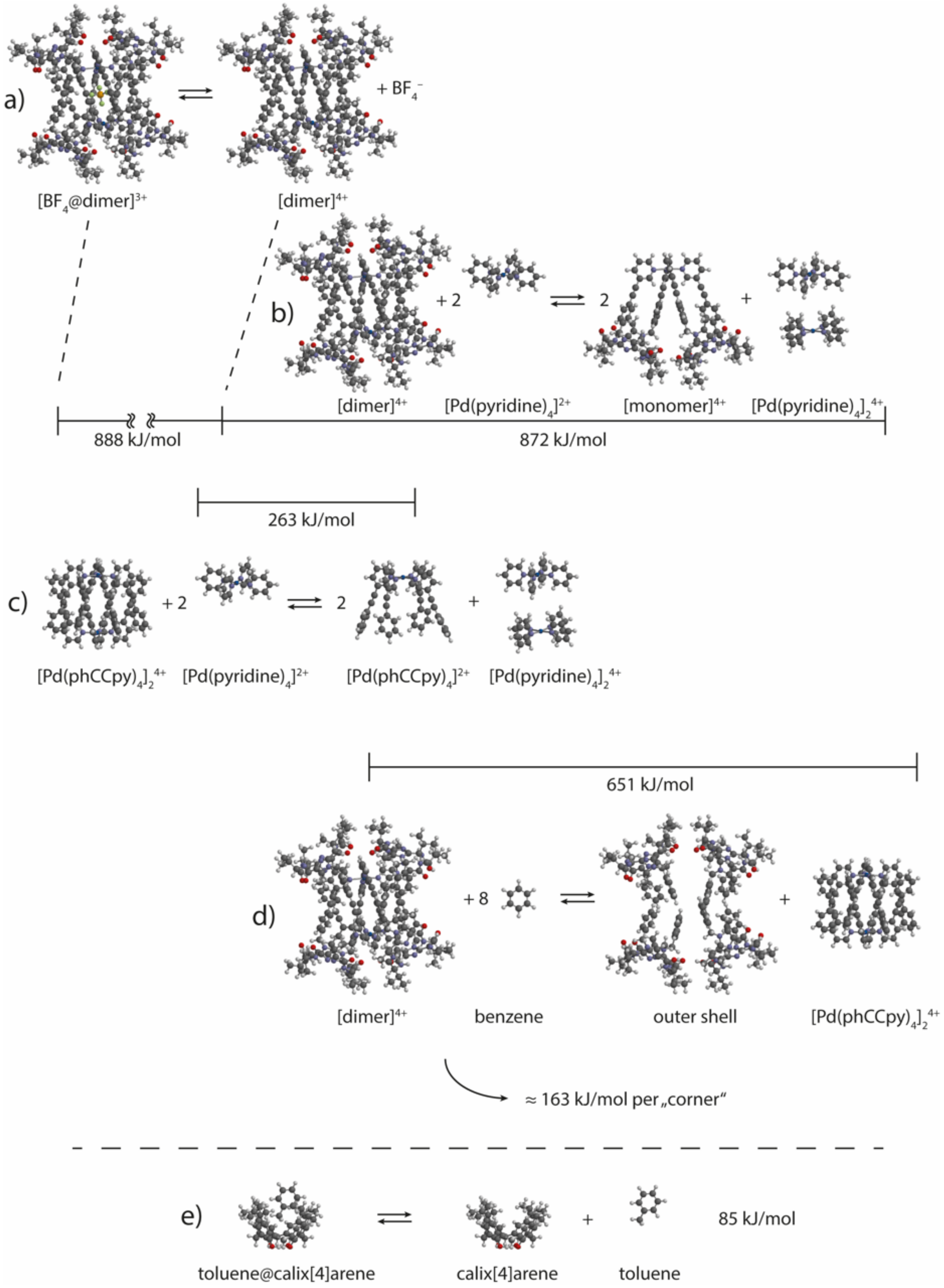

Figure S12. a)-d) Dissection of non-covalent contributions to the overall stability of the mechanically interlocked dimer (all values in kJ/mol from ωB97X-D/def2-TZVP gas-phase single point energy calculations on X-ray structure-derived fragments with PM6-otimized CH and XH bond lengths)

As a result, a major stabilizing contribution expectedly arose from binding the BF_4^- anion in the central cavity of the tetra-cationic assembly (Figure S12a), although care must be taken when comparing the herein described gas phase calculation results, only considering the encapsulated anion, with the real situation in polar organic solvent and in presence of further freely moving counter anions. After removing the central anion, we further dissected dimer stabilization in the following way: First, the energy difference in the formal dimer/monomer equilibrium was computed, corrected by the Coulomb repulsive contribution that arises through the close proximity of the di-cationic Pd centers in the dimeric species (Figure S12b), yielding 872 kJ/mol as sum of non-covalent contributions stabilizing the dimer over the monomer. This value was further separated into c) π-stacking contribution within the inner core of the structure, i.e. between the eight interlacing phenyl-alkynyl-pyridyl arms that coordinate the two palladium centers (again corrected for Coulomb repulsion), amounting to 263 kJ/mol and d) the interaction of the inner core with the outer shell, mainly consisting of the peptidic macrocycles capping the four corners of the assembly, thereby engulfing one pyridyl substituent of the respective other lemniscate, each, in a kind of host-guest interaction. This contribution, as calculated by a formal homodesmic reaction scheme shown in Figure S12d, was determined to be 651 kJ/mol, adding up with the former π-stacking value to 914 kJ/mol, not far from the overall non-covalent contribution calculated in a) to be 872 kJ/mol. Division by four resulted in a stabilization of 163 kJ/mol per "corner" of the inner core embedded into the four macrocycles. The structure of this host-guest interaction somehow resembles the classical example of the toluene@calix[4]arene complex, for which we computed a stabilization of 85 kJ/mol (same procedure: PM6-optimization of CH distances of its X-ray structure,[16] followed by ωB97X-D/def2-TZVP gas-phase single point energy calculations).

In order to showcase the degree of London dispersion contributions to the stabilization of tetra-cationic dimer $[Pd_2L_4]^{4+}$ over monomer $[PdL_2]^{2+}$, we repeated step b) with a method not considering empirical dispersion (wB97X/6-31G*-LanL2DZ) and obtained a stabilization reduced by about 300 kJ/mol as compared to the dispersion-corrected approach (ωB97X-D/6-31G*-LanL2DZ) with the same basis set.

4 Ion Mobility Measurements

Ion mobility measurements were performed on a Bruker timsTOF instrument combining a trapped ion mobility (TIMS) with a time-of-flight (TOF) mass spectrometer in one instrument.

In contrast to the conventional drift tube method to determine mobility data, where ions are carried by an electric field through a stationary drift gas, the TIMS method is based on an electric field ramp to hold ions in place against a carrier gas pushing them in the direction of the analyzer. Consequently, larger sized ions that experience more carrier gas impacts leave the TIMS units first and smaller ions elute later. This method offers a much higher mobility resolution despite a smaller device size.

Measurement: After the generation of ions by electrospray ionisation (ESI, analyte concentration: 0.1 mg/mL solvent: Acetonitrile, capillary voltage: 3600V, end plate offset voltage: 500V, nebulizer gas pressure: 0.3 bar, dry gas flow rate: 3.0 l/min, dry temperature: 200 °C) the desired ions were orthogonally deflected into the TIMS cell consisting of an entrance funnel, the TIMS analyser (carrier gas: N_2, temperature: 305 K, entrance pressure: 2.55 mbar, exit pressure: 0.89 mbar, IMS imeX ramp end: 1.91 1/K0, IMS imeX ramp start: 0.89 1/K0) and an exit funnel. As a result, the ions are stationary trapped. After accumulation (accumulation time: 40 ms), a stepwise reduction of the electric field strength leads to a release of ion packages separated by their mobility. After a subsequent focussing, the separated ions are transferred to the TOF-analyser.[17-19]

The ion mobility *K* was directly calculated from the trapping electric field strength *E* and the velocity of the carrier gas stream v_g *via*

$$K = \frac{v_g}{E} = \frac{A}{U_{release} - U_{out}} \tag{1}$$

where *A* is a calibration constant (based on calibration standards), $U_{release}$ is the voltage at which the ions are released from the analyser and U_{out} is the voltage applied to the exit of the tube. The ion mobility is corrected to standard gas density *via*

$$K_0 = K \frac{P}{1013\ \text{hPa}} \frac{237\ \text{K}}{T} \tag{2}$$

to obtain the reduced mobility K_0, where *P* is the pressure and *T* is the temperature. By using the Mason-Schamp equation, the collisional cross-section (CCS) Ω can be calculated:

$$\Omega = \frac{(18\pi)^{\frac{1}{2}}}{16} \frac{ze}{(k_B T)^{\frac{1}{2}}} \left[\frac{1}{\mu}\right]^{\frac{1}{2}} \frac{1}{K_0} \frac{1}{N_0} \tag{3}$$

where ze is the ion charge, k_B is the Boltzmann constant, μ is the reduced mass of analyte and carrier gas and N_0 is the number density of the neutral gas.

For calibration of both the TIMS and TOF analysers, commercially available Agilent ESI tuning mix was used. The instrument was calibrated before each measurement, including each change in the ion mobility resolution mode ("imeX" settings: survey, detect or ultra).

Obtained ion mobility curves as well as derived CCS values are depicted in Figure 3 in the main text.

5 References

1 Y. Kashiwagi, S. Chiba, H. Ikezoe, J.-i. Anzai, 2513; *Synlett* **2004**, 2513–2516.

2 G. Haberhauer, *Angew. Chem.* **2007**, *119*, 4476-4479; *Angew. Chem., Int. Ed.* **2007**, *46*, 4397–4399.

3 A. Burkhardt, T. Pakendorf, B. Reime, J. Meyer, P. Fischer, N. Stübe, S. Panneerselvam, O. Lorbeer, K. Stachnik, M. Warmer, P. Rödig, D. Göries and A. Meents, *Eur. Phys. J. Plus*, **2016**, *131*, 56.

4 W. Kabsch, *Acta Crystallogr. Sect. D*, **2010**, *66*, 125–132.

5 G. M. Sheldrick, *Acta Crystallogr. Sect. A*, **2015**, *71*, 3–8.

6 G. M. Sheldrick, *Acta Crystallogr. Sect. C*, **2015**, *71*, 3–8.

7 C. B. Hübschle, G. M. Sheldrick and B. Dittrich, *J. Appl. Cryst.*, 2011, **44**, 1281–1284.

8 D. Kratzert, J. J. Holstein and I. Krossing, *J. Appl. Cryst.*, **2015**, *48*, 933–938.

9 D. Kratzert, I. Krossing, *J. Appl. Cryst.,* **2018**, 51, 928-934.

10 A. Thorn, B. Dittrich and G. M. Sheldrick, *Acta Crystallogr. Sect. A*, **2012**, *68*, 448–451.

11 A. Spek, *Acta Crystallogr. Sect. C*, **2015**, *71*, 9–18.

12 A. Spek, *Acta Crystallogr. Sect. D*, **2009**, *65*, 148–155.

13 Spartan `18 Parallel Suite, Wavefunction, Inc., Irvine

14 J. G. Brandenburg, C. Bannwarth, A. Hansen, S. Grimme, *Chem. Phys.* **2018**, *148*, 064104.

15 F. Neese, *WIREs Comput. Mol. Sci.* **2017**, e1327.

16 A. Arduini, R. Caciuffo, S. Geremia, C. Ferrero, F. Ugozzoli, F. Zontone, *Supramolecular Chemistry* **1998**, *10*, 125-132.

17 F. A. Fernandez-Lima, D. A. Kaplan, M. A. Park, *Rev. Sci. Instrum.* **2011**, *82*, 126106.

18 D. R. Hernandez, J. D. DeBord, M. E. Ridgeway, D. A. Kaplan, M. A. Park, F. Fernandez-Lima, *Analyst* **2014**, *139*, 1913–1921.

19 J.-F. Greisch, J. Chmela, M. E. Harding, D. Wunderlich, B. Schäfer, M. Ruben, W. Klopper, D. Schooss, M. M. Kappes, *Phys. Chem. Chem. Phys.* **2017**, *19*, 6105–6112.
